# Effects of aerobic training with and without weight loss on insulin sensitivity and lipids

**DOI:** 10.1371/journal.pone.0196637

**Published:** 2018-05-18

**Authors:** Damon L. Swift, Joseph A. Houmard, Cris A. Slentz, William E. Kraus

**Affiliations:** 1 Department of Kinesiology, East Carolina University, Greenville, NC, United States of America; 2 Duke University School of Medicine, Durham, NC, United States of America; Weill Cornell Medical College in Qatar, QATAR

## Abstract

**Purpose:**

The purpose of this study is to evaluate the effect of exercise training with modest or greater weight loss (≥3%) or not (<3%) on insulin sensitivity, lipoprotein concentrations, and lipoprotein particle size in overweight and obese participants.

**Methods:**

Adults (N = 163, body mass index: 25–37 [kg/m^2^]) participated in 8 months of exercise training. Insulin sensitivity, lipid concentrations, lipid particle size and other cardiometabolic variables were measured at baseline and follow-up. Participants were categorized by whether they achieved at least modest weight loss (≥ 3%) or not (<3%) following the intervention.

**Results:**

A greater improvement in insulin sensitivity was observed in adults performing exercise training with at least modest weight loss (2.2 mU·l^-1^ ·min ^-1^, CI: 1.5 to 2.8) compared to those who did not (0.8 mU·l^-1^ ·min ^-1^, CI: 0.5 to 1.2). Similar results were observed for acute insulin response, triglycerides, non-HDL cholesterol concentration, low density lipoprotein (LDL) particle size and high density lipoprotein (HDL) particle size (p<0.05), when all exercise groups were combined. No significant results across weight loss categories were observed for LDL, HDL, glucose, or insulin levels.

**Conclusion:**

The present study suggests that aerobic exercise combined with at least modest weight loss leads to greater improvements in insulin sensitivity, triglycerides as well as other non-traditional lipid risk factors (non-HDL cholesterol, HDL/LDL particle size). Clinicians should advocate patients who are overweight/obese to exercise and obtain modest weight loss for improved cardiovascular benefits.

## Introduction

Exercise and weight loss are recommended strategies to improve cardiovascular health [1, 2]. Improvements in risk factors for cardiovascular disease occur in individuals who obtain as little as 2–3% weight loss, as well as those that have achieved clinically significant weight loss (≥5%) [3–6]. However, the weight-independent and the weight-dependent cardiometabolic benefits in individuals who complete an exercise training program have not been fully resolved. Many of the previous studies in this area have compared the cardiometabolic responses in diet versus exercise-induced weight loss [7, 8] or by the mean weight loss of the overall study sample [4, 9, 10]. Thus, little work has evaluated the responses of cardiometabolic risk factors in exercisers losing a meaningful amount of weight (≥ 3%) compared to exercisers that do not. Given the large magnitude of heterogeneity in weight loss responses following an exercise training program in overweight/obese adults [11], this distinction is clinically important. More information in this area may help elucidate the clinical relevance of weight loss targets in actively exercising individuals and establish clinical cut-points/goals for sedentary overweight and obese patients.

Recently, our group [3] reported that compared to participants who did not lose a significant amount of weight with exercise (≤3%), modest weight loss (defined between 3% and 4.9% weight loss) or clinically significant weight loss (≥5.0%) was necessary to improve insulin sensitivity (assessed by homeostatic model of insulin resistance [HOMA-IR]. Additionally, no weight loss/exercise interactions were evident for other cardiometabolic risk factors (e.g. lipids, glucose, absolute fitness). Importantly in that study, we did not assess a dynamic measure of insulin action (HOMA-IR measures steady-state glucose homeostasis), only evaluated moderate intensity exercise training and studied only postmenopausal women. Importantly, to our knowledge, no study has evaluated the effect of modest weight loss in conjunction with exercise training on non-traditional risk factors such as changes in non-HDL cholesterol, lipoprotein particle size/number, which may have prognostic value above lipid concentrations [12–14].

The purpose of the present study is to evaluate the effect of aerobic exercise training in those who have achieved modest weight loss (≥3%) compared to those that did not (<3%) on insulin sensitivity as assessed by an intravenous glucose tolerance test, and on lipoprotein concentrations, particle size, and non-HDL cholesterol.

## Methods

Data for the present study were obtained from the STRRIDE study. The full methodology [15] and the main outcomes [15–17] of STRRIDE have been previously reported. Briefly, STRRIDE was a multi-center trial evaluating the effect of exercise intensity and amount on cardiometabolic risk factors. Data for the STRRIDE study was obtained between 1998–2002. The study included overweight/obese (BMI: 25–37 [kg/m^2^]) sedentary adults aged 40–65 years with mild to moderate dyslipidemia. Moderate dyslipidemia was defined as elevated low density lipoprotein (LDL) levels (130 to 190 mg/dL) or depressed high density lipoprotein values (men: <35 mg/dL, women: <45 mg/dL). Sedentary status was defined as participation in exercise less than once a week and a peak oxygen consumption value below 35 mL O_2_•kg^-1^•min ^-1^. The exclusion criteria for study participation included a fasting glucose level >140 mg/dL, excessively high systolic (>160 mmHg) or diastolic (>90 mmHg) blood pressure (blood pressure medication was not permitted). In addition, individuals were ineligible if they had overt coronary heart disease, metabolic/musculoskeletal disorders, currently engaged in dieting, and unwillingness to participate in all aspects of the research protocol. All women included in the STRRIDE study were postmenopausal, which was confirmed by measurement of serum follicle stimulating hormone levels (>40 UI/L). Written informed consent was obtained from all participants. The protocol was reviewed and approved annually by the Duke University and East Carolina University institutional review boards.

### Outcome measurements

Outcome measures were collected at baseline and after the completion of the exercise program. Body composition was determined by skin fold calipers [18]. Waist circumference was measured at the minimal waist and the umbilicus. Maximal oxygen consumption was measured using an incremental treadmill test with a 12-lead electrocardiogram. Participants exercised until volitional exhaustion. Expired respiratory gases were measured continuously using a Sensor Medics model 2900 and Vmax U (Yorba Linda, CA) or a TrueMax 2400 (Parvomedics, Sandy, UT). Fitness measures were quantified in relative (mL O_2_•kg^-1^•min ^-1^) and absolute VO_2_ peak (L O_2_/min).

### Intravenous glucose tolerance test

Insulin sensitivity was assessed using an intravenous glucose tolerance test (IVGTT). After the collection of fasting blood samples, glucose (50%) was injected into a catheter placed in the antecubital vein at a dose of 0.3 g/kg body weight. Subsequently, blood samples were obtained at the following time points: 2, 3, 4, 5, 6, 8, 10, 12, 14, 16, 19, 22, 25, 30, 40, 50, 60, 70, 80, 90, 100, 120, 140, 160, and 180 min. Insulin was injected at minute 20 of the test at a dose of 0.025 U/kg body mass. Blood samples were centrifuged, and stored at -80°C until sample analysis for glucose and insulin. Glucose levels were measured using a YSI model 2300 Stat Plus (Yellow Springs Instruments, OH). Insulin levels were measured using Access Immunoassay System (Beckman Coulter, Fullerton, CA). An insulin sensitivity index was determined through a minimal model [19]. IVGTT data were obtained approximately 24 hours [17] following the last exercise training session.

### Lipid measures

Nuclear magnetic resonance spectroscopy was used to determine lipoprotein low density lipoprotein [LDL], high density lipoprotein [HDL] particle size and estimates of LDL, HDL, total cholesterol and triglyceride concentrations. Non-HDL cholesterol was determined by subtracting HDL cholesterol from total cholesterol.

### Nutritional data

Upon enrolling in the STRRIDE study, participants were trained on how to properly report nutritional information about food composition and portion size. Nutritional data in the present analysis were obtained at baseline and follow-up. Participants completed 3-day dietary (3-DDR) records about all food and beverages ingested over 3 total days (one of which was a weekend day). STRRIDE staff also called participants to complete a 24-hour food recall (24-HrDR) in an announced phone call. During this call, study staff first asked participants to report all the food/beverages consumed over the last 24-hours. Following this, staff asked probing questions to assure the participants had reported everything they had consumed. Staff then asked about the time and occasion for each item consumed. Last, staff reviewed the list with the participant for accuracy and to prompt the memory of any unreported items. The use of a multiple pass approach for 24-HrDR reduces under-reporting and allows for opportunities to enhance the overall recall of nutritional items. Data from the 3-DDR and the 24-HrDR were analyzed using Food Processor Nutrition Analysis Software (version 7.1, 1996; ESHA 159Research, Salem, OR). Nutritional data from the 3-DDR and the 24-HrDR were averaged. For the present paper, total caloric intake and percent of total calories from carbohydrates, protein and fats are reported.

### Randomization

Following the completion of baseline measures, participants were randomized to the low amount, moderate intensity (LAMI), low amount, high intensity (LAHI), high amount, high intensity (HAHI), or to a non-exercise control group. The randomization procedure for STRRIDE has been previously described [15]. Briefly, the randomization for participants was performed using a randomized block design and were computer generated. Separate sets of randomization blocks were generated for each study site (East Carolina University and Duke University). Within each site, randomization blocks were created for white women, minority women, white men and minority men [15].

### Exercise training

Participants randomized to the exercise group participated in 8 months of supervised aerobic exercise training. The LAMI group trained at an exercise dose of 14 kilocalories per kg per week (KKW) at an intensity of 40%-55% of peak oxygen consumption. The LAHI group trained at an exercise dose of 14 KKW and at an intensity of 65%-85% of peak oxygen consumption. The HAHI group trained at an exercise dose of 23 KKW and at an intensity of 65%-85% of peak oxygen consumption. Participants were permitted to exercise using a stationary ergometer, treadmill (with a 3% grade), stairmaster, or elliptical trainers.

### Weight loss categorization

Participants were categorized on whether or they achieved at least modest (≥3%) or did not (<3%) following the intervention. The categorization for modest weight loss based on data suggesting that weight loss as little as 2–3% is associated with cardiovascular benefits [3–5, 20, 21].

### Sample size

The primary purpose of the present analysis was to evaluate the additive effects of habitual exercise training and weight loss on insulin action. Therefore, from the full sample of participants in the STRRIDE database (N = 387), we excluded individuals that did not complete the study (n = 127), participants in the control group (n = 61) (only 4 participants in the control group achieved modest weight loss), exercisers who had inadequate exercise training adherence levels (<70%) (n = 19) and exercisers who did not have intravenous glucose tolerance test measurements at both baseline and follow-up (n = 17). Thus, a total of 163 were included in the final analysis. Of the participants that met the criteria for this analysis, there were 53, 54, and 56 participants that were randomized to the LAMI, LAHI and HAHI groups, respectively.

### Statistical procedure

Statistical analyses were conducted using SAS version 9.3 (Cary, NC). Continuous baseline data were summarized as means (± standard deviation [SD]) and were compared across randomization groups (i.e. LAMI, LAHI, HAHI) through a one-way analysis of variance. Baseline variables with non-normal distributions were run with a Kruskal Wallis tests. Categorical baseline data was summarized in frequencies (%) and compared across randomization groups using a chi-square test. Baseline data were compared in the same manner for participants who achieved modest weight loss compared to participant that did not.

An analysis of covariance (ANCOVA) was used to compare the changes in cardiometabolic variables among those that achieved at least modest weight loss and those that did not following exercise training (within each exercise training group) with baseline value entered as a covariate. In addition, we performed ANCOVAs to evaluate the change in cardiometabolic risk factors in all exercisers groups combined to evaluate the general effect of aerobic training (when exercise dose/intensity were adjusted) with baseline value and exercise group (LAMI, LAHI, and HAHI) entered as a covariate in the model. For several lipid variables, sex was found to be a significant covariate (change in HDL, LDL, total cholesterol, large HDL particles, HDL size, and LDL size), therefore the ANCOVA models were additionally adjusted for sex. Similarly, race was found to be a significant covariate for change in AIRG and the ANCOVA model was additionally adjusted for race. The results of all ANCOVAs are presented in adjusted least squared means with 95% confidence intervals.

## Results

Baseline characteristics from the present study across exercise groups are shown in Table 1. The sample had a mean (SD) age of 52.4 (6.4) years, a BMI of 29.7 (2.8) kg/m^2^ and was 13.5% African American. No significant differences were observed for continuous or categorical variables at baseline across groups. Demographic data for participants stratified across whether or not they achieved at least modest weight loss are shown in Table 2. No significant differences were observed for continuous or categorical variables (all, p<0.05).

**Table 1:** Baseline participant characteristics.

	All Groups	LAMI	LAHI	HAHI
Variable	(N = 163)	(n = 53)	(n = 54)	(n = 56)
Age (yrs.)	52.4 (6.4)	53.6 (5.7)	52.0 (7.2)	51.8 (6.2)
Female n (%)	71 (43.6)	25 (47.2)	23 (42.6)	23 (41.1)
Race				
Caucasian n (%)	136 (83.4)	44 (83.0)	43 (79.6)	49 (87.5)
African American n (%)	22 (13.5)	9 (16.9)	9 (16.7)	4 (7.1)
Hispanic n (%)	2 (1.2)	0 (0.0)	0 (0.0)	2.0 (3.5)
Asian n (%)	3 (1.8)	0 (0.0)	2 (3.7)	1 (1.8)
BMI (kg/m^2^)	29.7 (2.8)	29.5 (2.8)	30.0 (2.9)	29.7 (2.8)
Weight (kg)	87.5 (13.8)	86.2 (15.2)	88.1 (12.4)	88.3 (13.8)
Waist circum. (natural) (cm)	95.8 (10.0)	95.2 (11.3)	95.3 (8.3)	96.8 (10.5)
Waist circum. (umbilicus) (cm)	103.1 (9.8)	102.3 (11.2)	103.3 (9.3)	103.6 (9.1)
Glucose (mg/dL)	93.6 (9.2)	93.5 (8.9)	93.1 (8.6)	94.1 (10.0)
Insulin (mIU/L)	9.3 (6.1)	9.7 (7.2)	8.7 (5.7)	9.4 (5.4)
HOMA-IR	2.2 (1.5)	2.3 (1.7)	2.1 (1.5)	2.2 (1.4)
Insulin sensitivity (mU·l^-1^ ·min ^-1^)	3.4 (2.4)	3.2 (2.2)	3.8 (2.2)	3.3 (2.6)
AIRg, (mU·l^-1^·min^-1^)	461.5 (371.6)	427.7 (302.5)	438.3 (359.4)	515.8 (437.1)
Sg (per min)	0.0187 (0.008)	0.019 (0.009)	0.019 (0.008)	0.018 (0.007)
Low density lipoprotein (mg/dL)	126.8 (24.3)	124.1 (20.7)	130.3 (27.1)	125.6 (24.4)
High density lipoprotein (mg/dL)	46.2 (14.3)	47.4 (14.2)	45.4 (13.2)	45.8 (15.7)
Total cholesterol (mg/dL)	197.9 (31.1)	197.4 (28.7)	199.3 (32.9)	196.9 (31.7)
Triglycerides (mg/dL)	157.7 (90.0)	170.0 (110.6)	143.9 (67.8)	160.4 (88.5)
Non-HDL cholesterol (mg/dL)	8.8 (0.4)	150.0 (23.5)	153.9 (30.6)	151.1 (25.8)
Small LDL cholesterol (mg/dL)	38.3 (34.0)	37.2 (33.8)	42.6 (34.7)	35.0 (33.8)
Large HDL cholesterol (mg/dL)	7.6 (7.1)	7.5 (5.7)	7.9 (8.1)	7.4 (7.2)
LDL particles (mmol/L)	1420.8 (346.3)	1381.3 (287.6)	1450.6 (378.4)	1427.2 (364.8)
LDL particle size (nm)	20.8 (0.9)	20.8 (0.9)	20.8 (0.8)	20.7 (1.0)
HDL particle size (nm)	8.8 (0.4)	8.9 (0.4)	8.9 (0.4)	8.8 (0.4)
VO_2_ max (ml^.^kg^.^min)	28.3 (5.8)	27.3 (5.7)	29.3 (6.2)	28.1 (5.3)
VO_2_ max (L/min)	2.5 (0.7)	2.4 (0.7)	2.6 (0.8)	2.5 (0.7)

Continuous variables are expressed in mean (SD) and categorical variables are expressed n (%). BMI: Body mass index, AIRg: Acute insulin response to glucose, Sg: Glucose effectiveness, Non-HDL cholesterol: Non high density lipoprotein cholesterol, Small LDL cholesterol: Small low density lipoprotein particles,

LDL particles: Total low density lipoprotein particles, VO_2_ max: maximal oxygen consumption

**Table 2:** Baseline characteristics, percent weight loss and interventional data in participants who achieved at least modest weight loss (≥ 3%) following the intervention compared to those that did not (<3%).

Variable	No Weight loss (n = 126)	Weight loss (n = 37)
Age (yrs.)	52.3 (6.4)	52.9 (6.6)
Female (%), n	44.4 (56)	40.5 (15)
Race		
Caucasian n (%)	104 (82.5)	32 (86.5)
African American n (%)	14.3 (18)	10.8 (4)
Hispanic n (%)	2 (1.6)	0 (0)
Asian n (%)	2 (1.6)	1 (0.6)
Weight (kg)	87.7 (13.8)	87.0 (14.0)
%Weight loss	-0.9 (1.9)	-5.3 (2.0) *
Waist circumference (natural) (cm)	95.7 (10.1)	96.1 (10.0)
Waist circumference (umbilicus) (cm)	103.3 (10.1)	102.4 (9.1)
Glucose (mg/dL)	93.8 (9.5)	92.7 (7.9)
Insulin (mIU/L)	9.5 (6.3)	8.6 (5.7)
HOMA-IR	2.2 (1.5)	2.0 (1.5)
Insulin sensitivity (mU·l^-1^ ·min ^-1^)	3.3 (2.2)	3.9 (2.8)
AIRg (mU·l^-1^·min^-1^)	459.6 (363.6)	468.0 (402.8)
Sg (per min)	0.018 (0.008)	0.020 (0.008)
Low density lipoprotein (mg/dL)	126.4 (24.4)	128.0 (24.0)
High density lipoprotein (mg/dL)	46.3 (14.3)	45.6 (14.4)
Total cholesterol (mg/dL)	197.7 (31.5)	198.5 (30.0)
Triglycerides (mg/dL)	156.5 (89.1)	161.6 (94.4)
Non-HDL cholesterol (mg/dL)	151.4 (27.6)	153.0 (23.7)
Small LDL cholesterol (mg/dL)	38.1 (33.9)	39.2 (35.0)
Large HDL cholesterol (mg/dL)	7.7 (7.4)	7.3 (5.7)
LDL particles (mmol/L)	1411.7 (348.7)	1453.2 (341.0)
Low density lipoprotein particle size (nm)	20.8 (0.9)	20.7 (0.9)
High density lipoprotein particle size (nm)	8.9 (0.4)	8.8 (0.4)
VO_2_ max (ml^.^kg^.^min)	28.4 (5.9)	27.8 (5.2)
VO_2_ max (L/min)	2.5 (0.7)	2.5 (0.7)
Exercise time (minutes per week)	174.4 (86.0)	181.1 (92.0)
Exercise adherence (%)	88.9% (0.7)	95.2% (0.8) *

Continuous variables are expressed in mean (SD) and categorical variables are expressed in (%), n

* Indicates significant difference compared to No weight loss condition. AIRg: Acute insulin response to glucose, Sg: Glucose effectiveness

The mean percent weight loss following exercise training was 0.9%, 1.0% and 1.9% in the LAMI, LAHI and HAHI groups, respectively. The prevalence of modest weight loss (3% to 4.9%) was 18.9%, 16.7% and 32.1% in the LAMI, LAHI, and HAHI groups, respectively. The prevalence of clinically significant weight loss (>5.0% weight loss from baseline) was 7.6%, 9.3%, and 14.3% in the LAMI, LAHI, and HAHI groups, respectively. No significant differences were observed across groups in the prevalence of modest weight loss (p = 0.110) or clinically significant weight loss (p = 0.486). However, higher levels of exercise adherence was observed for those that achieved modest weight loss compared to those that did not (p = 0.001).

Table 3 shows the changes in body composition, glucose homeostasis and fitness in participants with at least modest weight loss and those that did not. Larger changes in weight, waist circumference and relative fitness were observed in participants that achieved at least modest weight loss in all exercise groups (all p-values <0.05) compared to those that did not. In the HAHI group, a larger increase in relative fitness was evident in those with at least modest weight loss compared to those that did not. The change in fasting insulin and HOMA-IR approached significance in those that achieved modest weight loss compared to those who did not in the LAHI and the HAHI groups (p-values between 0.05 and 0.09). In addition, a reduction in the acute insulin response (AIRg) was observed in the participants that had at least modest weight loss compared to those that did not in the HAHI group (approached significance for the LAHI group). No significant results for AIRg was observed in the LAHI group.

**Table 3:** The effect of exercise training with at least modest weight loss (≥3%) compared to those that did not (<3%) on body composition, insulin resistance and fitness An ANCOVA with adjustment for the baseline values was used to compare the change in these variables between the weight loss conditions.

	LAMI		LAHI		HAHI		All Exercise groups	
	No WL (n = 43)	MWL (n = 10)	No WL (n = 45)	MWL (n = 9)	No WL (n = 38)	MWL (n = 18)	No WL (n = 126)	MWL (n = 37)
Percent weight loss (%)	0.0	-5.0 *	-0.1	-5.2 *	-0.2	-5.5*	-0.1	-5.2 *
Δ Weight (kg)	0.1	-4.1 *	-0.1	-4.4 *	-0.2	-4.9 *	-0.1	-4.5 *
Δ BMI (kg/m^2^)	0.2	-1.5 *	-0.4	-1.6 *	-0.04	-1.6 *	-0.02	-1.6 *
Δ Waist circumference (natural) (cm)	0.1	-4.1 *	-0.1	4.4 *	-0.2	-4.9 *	-1.1	-3.9 *
Δ Waist circumference (umbilicus) (cm)	-0.6	-4.1 *	-1.2	-3.6*	-1.2	-5.5 *	-1.0	-4.4 *
Δ Glucose (mg/dL)	-0.3	2.3	1.5	-1.3	0.8	-0.3	0.7	0.2
Δ Insulin (mIU/L)	-1.7	-1.1	-1.2	-3.2 †	-0.9	-2.5 †	-1.3	-2.3 †
Δ AIRg (mU·l^-1^·min^-1^)	-2.1	-50.8	-21.4	-126.4	0.9	-127.1*	-6.0	-101.5*
Δ Sg (per min)	0.0002	0.0030	-0.0020	-0.0002	0.0002	0.0029	-0.0005	0.0019
Δ HOMA-IR	-0.4	-0.1	-0.3	-0.74 †	-0.12	-0.56 †	-0.27	-0.48
Δ VO_2_ max (L/min)	0.13	0.25	0.29	0.21	0.37	0.42	0.27	0.30
Δ VO_2_ max (ml.kg.min)	1.4	4.2 *	3.3	4.6	4.1	6.3*	2.9	5.0 *

*Indicates significant difference between the change in the variable between no weight loss (<3%) and weight loss and weight loss (≥3%) conditions within the exercise group;

† Indicates that the difference between the change in the variable between no weight loss and weight loss condition approached significance (p value is between 0.05 to 0.09) within the exercise group.

NO WL: no weight loss, MWL: at least modest weight loss, BMI: Body mass index, AIRg: Acute insulin response to glucose, Sg: Glucose effectiveness, VO_2_ max: maximal oxygen consumption

The changes in lipid variables in participants who achieved at least modest weight loss following exercise training and those that did not are shown in Table 4. When all exercise groups were combined, larger reductions in triglycerides, non-HDL cholesterol and LDL particles were observed in participants that achieved at least modest weight loss compared to participants that did not. In the LAMI group, a larger reduction in triglycerides was evident in participants with modest weight loss compared to participants who did not. In the LAHI group, a larger change in triglycerides was observed in participants that achieved at least modest weight loss compared to participants who did not. No significant differences were evident based on weight loss for LDL, HDL, total cholesterol, or absolute fitness levels (all p-values> 0.05).

**Table 4:** The effect of exercise training with at least modest weight loss (≥3%) compared to those that did not (<3%) on lipid variables.

	LAMI		LAHI		HAHI		All Exercise groups	
	No WL (n = 43)	MWL (n = 10)	No WL (n = 45)	MWL (n = 9)	No WL (n = 38)	MWL (n = 18)	No WL (n = 126)	MWL (n = 37)
Δ Low density lipoprotein (mg/dL)	0.3	-6.6	3.0	0.5	0.1	-6.9	1.1	-4.3 †
Δ High density lipoprotein (mg/dL)	0.4	4.4 †	1.1	1.4	3.4	3.3	1.6	3.0
Δ Total cholesterol (mg/dL)	-0.8	-4.9	3.6	-2.2	2.8	-6.3	1.9	-4.5
Δ Triglycerides (mg/dL)	-24.9	-49.6	-14.2	-54.8 *	-24.3	-28.5	-20.1	-44.3 *
Δ Non-HDL cholesterol (mg/dL)	-1.5	-9.9	2.3	-3.9	-0.9	-10.2 †	-0.3	-8.0 *
Δ Small LDL cholesterol (mg/dL)	-5.0	-17.2	-10.9	-34.7 †	-11.4	-16.2	-9.1	-22.7 †
Δ Large HDL cholesterol (mg/dL)	0.6	3.4	-0.1	0.6	1.1	1.9	0.6	1.9 †
Δ LDL particles (mmol/L)	9.7	-148.9	18.4	-102.2	-39.3	-93.0	-3.7	-114.7 *

* Indicates significant difference between the change in the variable between no weight loss (<3%) and weight loss and weight loss (≥3%) conditions within the exercise group;

† Indicates that the p-value between the between the no weight loss and weight loss condition is between 0.05 to 0.09) within the exercise group.

NO WL: no weight loss, MWL: at least modest weight loss, Non-HDL cholesterol: Non high density lipoprotein cholesterol, Small LDL cholesterol: Small low density lipoprotein particles,

LDL particles: Total low density lipoprotein particles

**Fig 1:**
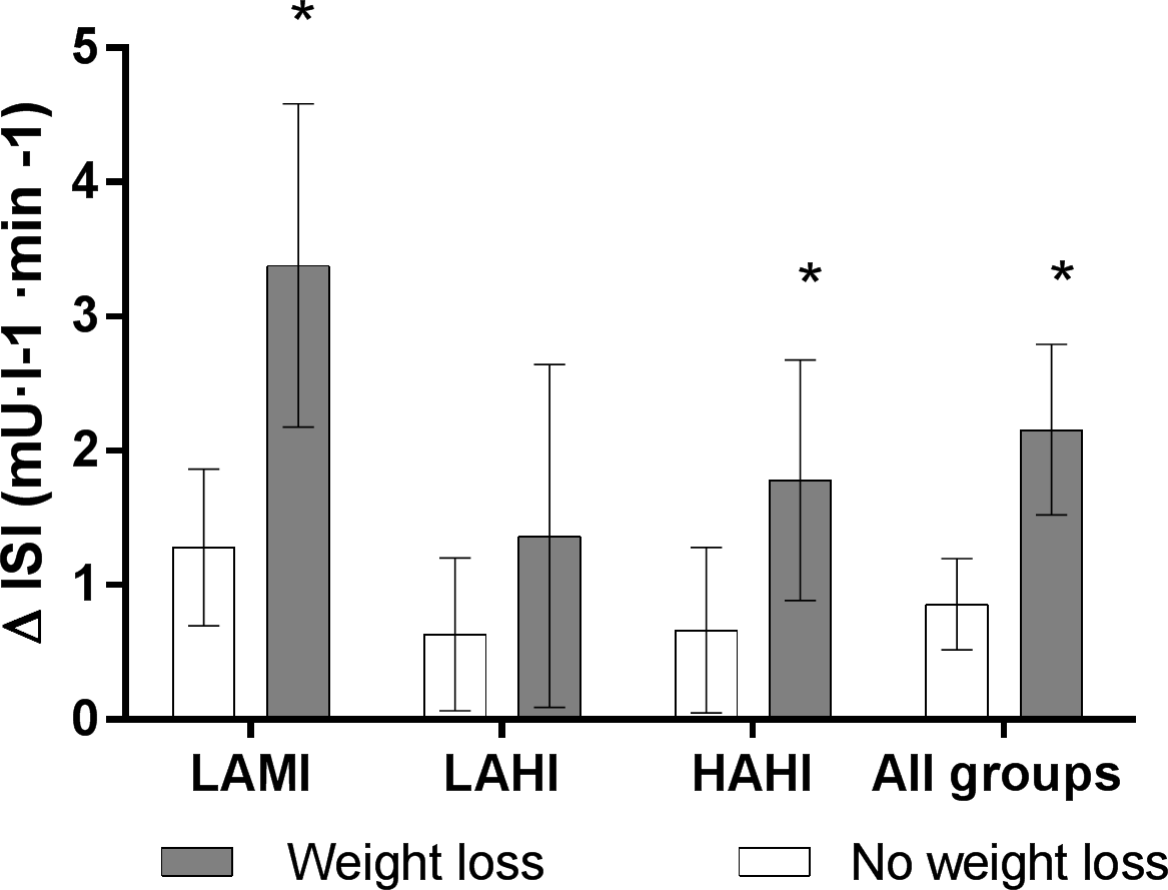
The effect of exercise training on insulin sensitivity in participants with at least modest weight loss (≥3%) and those that did not (<3%). *significant difference compared to no weight loss condition. LAMI: Low amount/low intensity, LAHI: Low amount/high intensity, HAHI: High amount/high intensity.

The changes in insulin sensitivity based on weight loss are shown in Fig 1. A significant within groups increase in insulin sensitivity was observed regardless of weight loss (all p-values<0.05). However, a larger increase in insulin sensitivity was observed in participants that achieved at least modest weight loss compared to those that did not in the LAMI (p = 0.002) and HAHI (p = 0.045) groups, but not the LAHI group (p = 0.340). When all groups were combined, a larger improvement in the change in insulin sensitivity (p = 0.006) was observed in those with at least modest weight loss compared to those that did not.

**Fig 2:**
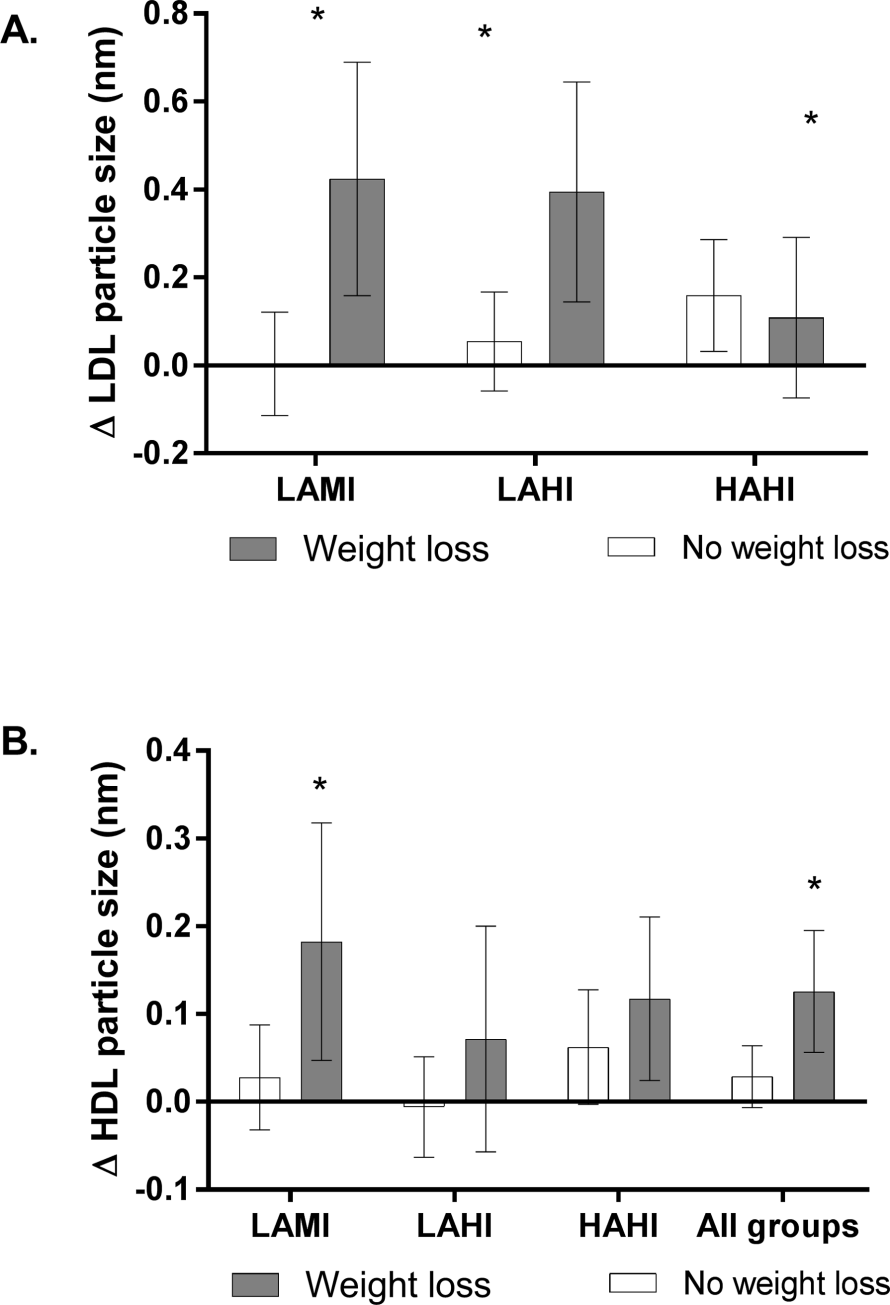
The effect of exercise training on LDL (Panel A) and HDL particle size (Panel B) in participants who achieved at least modest weight loss (≥3%) and those that did not (<3%). Note: LDL particle size with all groups combined was not run due to an interaction between change in LDL particle size and exercise group. *significant difference compared to no weight loss condition. LAMI: Low amount/low intensity, LAHI: Low amount/high intensity, HAHI: High amount/high intensity.

The change in lipoprotein particle size is shown in participants that achieved at least modest weight loss compared to those that did not is shown in Fig 2. A significant increase in LDL particle size (panel A) was observed in the in LAMI (p = 0.005) and LAHI (p = 0.015) groups in participants that achieved at least modest weight loss compared to that did not, but not the HAHI (p = 0.652) group. Exercise groups were not collapsed due to an interaction between exercise group and change in LDL particle size (p = 0.017). A significant increase in HDL particle size was observed in those with at least modest weight loss compared to those that did not in the LAMI (p = 0.040), but not the LAHI (p = 0.240), and HAHI (p = 0.354) groups. When all exercise groups were combined a larger improvement in HDL particle size (p = 0.015) in those with modest weight loss compared to those that did not.

Dietary information at baseline and after the exercise training is shown in Table 5. No significant differences were observed in total caloric intake or percent of calories from fat, carbohydrates or protein at baseline. Similarly, no significant changes in dietary variables were observed after training.

**Table 5:** The effect of exercise training with at least modest weight loss (≥3%) compared to those that did not (<3%) on nutritional variables.

Baseline	LAMI		LAHI		HAHI		All Exercise groups	
	No WL	MWL	No WL	MWL	No WL	MWL	No WL	MWL
Baseline	(n = 43)	(n = 10)	(n = 45)	(n = 9)	(n = 38)	(n = 18)	(n = 126)	(n = 37)
Caloric intake (kcals/day)	2113.2 (635.7)	2051.7 (768.0)	2122.9 (692.0)	2037.6 (468.6)	2183.3 (555.2)	2188.3 (506.3)	2137.8 (629.7)	2080.6 (564.5)
Protein intake (%)	15.3 (3.4)	17.9 (7.1)	16.9 (3.5)	12.4 (3.7)	16.3 (5.4)	18.4 (6.2)	16.2 (4.1)	16.8 (6.3)
Carbohydrate intake (%)	50.0 (6.5)	46.8 (13.3)	47.2 (10.1)	54.7 (11.4)	47.1 (10.0)	45.6 (9.8)	48.1 (9.0)	48.1 (11.5)
Fat intake (%)	33.7 (6.1)	33.9 (10.4)	34.5 (6.9)	32.0 (9.1)	35.0 (7.5)	35.1 (7.3)	34.4 (6.8)	34.0 (8.5)
**Changes after exercise training**	**LAMI**		**LAHI**		**HAHI**		**All Exercise groups**	
	**No WL**	**MWL**	**No WL**	**MWL**	**No WL**	**MWL**	**No WL**	**MWL**
Δ Caloric intake (kcals/day)	-161.5	-118.6	-161.3	-230.1	-126.4	-40.7	-65.5	-129.8
Δ Protein intake (%)	1.0	-1.0	-0.1	1.3	-1.2	-0.2	-0.1	0.0
Δ Carbohydrate intake (%)	-2.5	1.5	-3.2	-4.7	-1.3	-2.0	-2.4	-1.8
Δ Fat intake (%)	0.1	1.0	2.4	1.0	2.5	2.8	1.7	1.6

## Discussion

The findings of the present study suggest that in general overweight and obese participants exercise training and achieving at least modest weight loss (≥ 3%) were associated with larger improvements in insulin sensitivity compared to those who did not. In addition, we observed that participants who achieved at least modest weight loss with regular exercise had improvements in plasma lipid variables including non-HDL cholesterol, HDL particle size, LDL particle size/number and triglyceride concentrations. Our results suggest that although adhering to exercise programs consistent with public health recommendations can promote cardiometabolic benefits, clinicians should advocate for their participants to also seek at least modest weight loss for enhanced improvements in risk factors for cardiovascular disease.

The participants in the present study categorized as achieving at least modest weight loss lost an average of about ~5% with a minimum threshold of 3%, which suggests adaptations can occur with a relatively small magnitude of weight loss. The present study adds to the literature on weight loss targets in participants that are exercising by evaluating insulin sensitivity with a dynamic measure of insulin sensitivity (IVGTT) and assessing the effects of weight loss with exercise training on non-traditional lipid risk factors such as particle size, particle number and non-HDL cholesterol. Our results are generally in concert with Swift et al. [3] who evaluated the impact of both clinically significant (≥ 5% from baseline) and modest weight loss (between 3% and 4.9% weight loss) on cardiometabolic risk factors in postmenopausal women who participated in 6 months of moderate intensity exercise training (50% of VO_2_ max). In this study, greater improvements in insulin action (HOMA-IR) were observed with modest weight loss and clinically significant weight compared to those that were unable to achieve either, which seemed to be mediated primarily by reductions in fasting insulin levels. In the present study, the improvements in fasting insulin and HOMA-IR compared to those that did not approached significance in the LAHI and HAHI groups, but was not significant in the LAMI group. However, we did observe enhanced improvements in insulin sensitivity (measured via IVGTT) in participants that achieved modest weight loss, which supports that weight loss along with exercise training improves insulin action above the effects of exercise alone.

In terms of plasma lipids, we did not observe any changes in most of the traditional risk factors including concentrations of LDL, HDL, and total cholesterol. The only exception was reductions of triglyceride levels with modest weight loss compared to those with those that did not (LAMI group and all exercise groups combined). However, an important finding of the present study is the effect of exercise training with modest weight loss on improving non-traditional lipid risk factors such as non-HDL cholesterol (when all exercise groups were combined). Elevated non-HDL concentrations are associated with CVD mortality [13, 14, 22] and represent the combined atherogenic lipoproteins of the lipid profile [23]. In addition, recent research suggests that they are more predictive of cardiovascular risk than LDL concentration [23–25]. Similarly, we observed improvements in other lipid variables such as LDL/HDL particle size and some evidence for a reduction in LDL particle number suggesting a less atherogenic lipid profile in exercisers achieving modest weight loss. Importantly, these beneficial adaptations in lipids were not observed in participants who did not achieve weight loss.

Other training studies have reported improvements in the aforementioned lipid risk factors following exercise training studies including triglycerides [26], LDL/HDL particle size [26–28] and LDL particle concentration [26] and non-HDL cholesterol [29], although data were not evaluated specifically for weight loss categories. Halverstadt et al. [26] evaluated the changes in lipids in response to 24 weeks of aerobic exercise training. To reduce potential confounding effects of weight, this study had a dietary protocol to keep participants weight stable and included changes in body fat/weight in statistical models. The study observed improvements in various components of the lipid profile including LDL/triglycerides concentrations, HDL particle size, LDL particle concentrations, and small LDL cholesterol independent of weight loss. Varady et al. [28] observed increases in HDL particle size, but not LDL particle size in obese adults after 12 weeks of exercise training at 60% of maximum heart rate (~5.0% weight loss). However, Arsenault et al. [30] did not observe improvements in HDL and LDL particle size in response to 6 months of moderate intensity exercise training in postmenopausal women with elevated blood pressure (1.6% mean reduction in weight). In the present study, we did not observe within groups improvements in any lipid variables in participants who did not achieve modest weight loss with the exception of triglycerides, which suggests that the aerobic training alone was not sufficient to improve many of the lipid variables we examined. Although some evidence certainly exists suggesting that exercise training independent of weight loss can enhance lipid parameters, weight loss in addition to exercise training likely improves the probability of favorable responses. From a clinical perspective, exercising with weight loss may be particularly important for overweight/obese patients with pre-diabetes or dyslipidemia.

In reference to cardiorespiratory fitness, there was a greater increase in relative fitness in participants that obtained at least modest weight loss compared to those that did not. This is likely driven by the weight loss categories of the present analysis as there were no differences in absolute fitness between the modest weight loss and no weight loss conditions. However, cardiorespiratory fitness in an independent predictor of cardiovascular disease [31, 32]. A recent meta-analysis from Kodama et al. [33] suggests that a 1 MET is associated with a 15% reduction in cardiovascular disease. The difference in the change in METs between participants who obtained modest weight loss and those that did not was 0.6 METs, which is associated with a 9% reduction in CVD risk.

Another novel feature of this study was evaluating the exercise intensity/dose relationships within weight loss categories. The LAMI group had the greatest increase in insulin sensitivity, which exceeded the change within the weight loss groups of both the LAHI and the HAHI groups. This may suggest that the low amount moderate exercise combined with weight loss may represent the optimal strategy for the improvement in insulin sensitivity. In addition, the LAMI group with weight loss was the only exercise group to improve in HDL particle size compared to those in the LAMI group with no weight loss. These results should be interpreted with some caution given that this is a retrospective analysis and the sample size within each weight loss category may have limited the ability to detect significant differences for some risk factors between weight loss categories. However, as evidenced by the pooled analysis on risk factors, weight loss appears to improve many cardiometabolic risk factors.

Overall, the participants included in the present study lost 1.3% of their baseline weight with 22.7% of the sample achieving at least modest weight loss and 10.4% of the sample achieving clinically significant weight loss. In addition, the prevalence of either modest or clinically significant weight loss was not significantly different across exercise groups. Thus, the clinical implications are that exercise prescriptions between (40–85% VO_2_ max) and a dose of 14 to 23 KKWs of aerobic training result in a similar prevalence of modest and clinically significant weight loss. The participants included in the present study were highly adherent (~90%) to exercise training and we removed participants that did not achieve at least 70% adherence from the analysis.

Our results are in concert with other large randomized controlled trials which have suggested that aerobic training programs are unlikely to produce clinically significant weight loss on average [3, 4, 20, 34, 35] unless they greatly exceed (e.g. 5 times a week of exercise, ≥ 400 kcals per session) the minimum physical activity guidelines [7, 10, 36]. Behavioral methods for compensation for weight loss include reduced non-exercise physical activity levels and increased caloric consumption in response to exercise training. In STRRIDE, non-exercise physical activity measurement (via accelerometery) was performed on a subset of participants from the Duke site only (n = 50) [37] and therefore we did not have sufficient data to evaluate for differences in non-exercise physical activity across weight loss groups. In addition, nutritional data did not reveal dietary differences between weight loss groups in the study. Thus, a limitation of this study is that we are unable to address the major factor which produced the weight loss.

The present study has several strengths. STRRIDE was a randomized controlled trial in which the exercise groups were supervised by research staff and had distinct doses/intensity of exercise which had an aerobic dose consistent with physical activity recommendations. In addition, we utilized IVGTT to measure insulin sensitivity, which is an improvement over previous studies which have used less sophisticated measures to assess insulin resistance. The present study also has several weaknesses. As mentioned earlier, the STRRIDE study collected non-exercise physical activity data in a sub-set of participants, which in was insufficient for the present analysis. Additionally, although the use of IVGTT is an improvement compared to the available data, a hyperinsulinemic euglycemic clamp is considered the gold standard for assessment of insulin sensitivity. Last, this analysis is retrospective and therefore our data are limited by the number of participants available for analysis in each weight loss category. This may have limited our ability to detect differences in certain variables among each exercise group.

The results of the present study suggest that in overweight/obese adults participating in exercise training, the achievement of at least 3% weight loss promotes greater improvements in insulin sensitivity compared to those that did not. In addition, participants that achieved modest weight loss in general had more favorable responses in traditional (triglycerides) and non-traditional (non-HDL cholesterol, LDL particle size/number, and HDL particle size). These findings have public health and clinical implications as they suggest that overweight/obese adults with dyslipidemia or insulin resistance should be encouraged to attempt to lose at least modest amounts of weight along with exercise training to maximize the overall improvements in indices of metabolic health.
